# Orthorexia and deprivation as ‘care’: how people with disordered eating understand care

**DOI:** 10.1186/2050-2974-3-S1-O31

**Published:** 2015-11-23

**Authors:** Connie Musolino, Megan Warin, Tracey Wade, Peter Gilchrist

**Affiliations:** 1 University of Adelaide, Australia; 2 Gender Studies and Social Analysis, University of Adelaide, Australia; 3 School of Psychology, Flinders University, Australia; 4 Psychiatrist in private practice, Australia

## 

This paper examines how contemporary understandings of health and care are engaged with by women with disordered eating. Based on findings from an Australian Research Council grant study, we explore the ways in which people align themselves with ‘healthy eating’ principles to legitimize their practices. Rather than always seeing their practices as a problem in need of intervention, many participants actively pursued and ‘tinkered with’ (Mol et al., 2010) their disordered eating as a form of self-care. We investigate how participants use the new food regime of orthorexia, as well as food choices and intolerances, as a normalised cover for restrictive diets. We argue that orthorexic practices entailing natural, medical and ethical concerns were successfully incorporated into participants' eating disorder repertoires. Participant's commentary on orthorexia reveals the vulnerability of people with disordered eating to the panoply of health and fitness advice circulating in contemporary society in which the pursuit of health and healthy lifestyles are at the centre of moral virtue, personhood and citizenship (Crawford, 1980). We demonstrate how these eating and body practices relate to an ethics of care that promotes moral virtues of hard work, purity and deprivation – all of which can ultimately lead to dangerous restrictive practices. Understanding how categories of health and care are understood and transformed by people with disordered eating has important implications for identifying people at all stages of help seeking.

